# Atypical Blistering Manifestation of Secondary Syphilis: Case Report and Review of Reported Cases

**DOI:** 10.3390/idr17060143

**Published:** 2025-11-18

**Authors:** Agnieszka Markiewicz, Aleksandra Skórka, Agnieszka Owczarczyk-Saczonek

**Affiliations:** 1 Department and Clinic of Dermatology, Sexually Transmitted Diseases and Clinical Immunology, University of Warmia and Mazury, 10-229 Olsztyn, Poland; agnieszka.owczarczyk@uwm.edu.pl; 2 Students Organization, Department and Clinic of Dermatology, Sexually Transmitted Diseases and Clinical Immunology, University of Warmia and Mazury, 10-229 Olsztyn, Poland

**Keywords:** syphilis, secondary syphilis, blistering diseases

## Abstract

**Background/Objectives:** Secondary syphilis typically presents with a non-pruritic maculopapular rash. However, vesicular and bullous manifestations are exceedingly rare in adults and may mimic autoimmune blistering diseases. The objective of this report is to describe atypical presentation of secondary syphilis with predominant vesiculobullous lesions and to emphasize the importance of including syphilis in the differential diagnosis of blistering skin diseases. **Methods:** We describe the case of a 46-year-old bisexual man with syphilis of unknown duration who presented with recurrent polymorphic skin eruptions, predominantly bullous and vesicular in nature. Clinical examination, serologic testing, and histopathologic evaluation were performed to establish the diagnosis. **Results:** Serologic tests confirmed active syphilis infection. A brief review of similar reported cases was conducted to highlight the clinical variability of vesiculobullous syphilis. **Conclusions:** Atypical vesiculobullous presentations of secondary syphilis pose significant diagnostic challenges and may be mistaken for autoimmune blistering disorders. Clinicians should maintain a high index of suspicion for syphilis in patients with polymorphic or blistering eruptions, particularly in those with risk factors for sexually transmitted infections. Awareness of these uncommon manifestations can facilitate timely diagnosis and appropriate treatment.

## 1. Introduction

Syphilis is a sexually transmitted infection caused by *Treponema pallidum*. Its protean manifestations have earned it the title “the great imitator”. According to the ECDC, syphilis is categorised as early (≤1 year) or late (>1 year) from infection onset [1,2], while the WHO defines late syphilis as occurring after two years [3].

The symptomatology of syphilis is highly diverse and can pose significant diagnostic challenges. A considerable number of patients remain asymptomatic or present with only mild symptoms during various stages of the disease, and it is often only laboratory test results that may suggest the diagnosis. Moreover, in the era of widely available advanced diagnostic methods, physical examination of the patient is often pushed into the background.

Young physicians have limited opportunities to encounter and learn its clinical manifestations during their medical training. Meanwhile, the diagnosis and treatment of syphilis have not undergone substantial changes for decades and continue to pose major challenges for clinicians. The disease should always be considered—not only in patients with clear clinical signs, but also in those with nonspecific or inconsistent symptoms, especially when other diagnoses cannot be confirmed.

Sir William Osler, regarded as the father of modern medicine, once said, “He who knows syphilis, knows medicine.”

Secondary syphilis, a systemic manifestation resulting from bacteriemia, typically occurs within the first year of infection—most often around eight weeks after the healing of the primary ulcer. Generalised symptoms, including lymphadenopathy, are common. Its hallmark is a symmetrical on both sides of the body, non-pruritic rash, frequently involving the palms and soles. The initial lesions usually present as a macular rash, while recurrent episodes more commonly manifest as maculopapular or pustular eruptions. Recurring lesions are not symmetrical and have the tendency to group into clusters [4,5,6,7]. Numerous authors have reported atypical manifestations of secondary syphilis [7,8,9,10,11,12]. In rare cases, vesicular or bullous lesions may occur, necessitating differentiation from autoimmune blistering diseases.

After approximately 4 to 12 weeks from the onset of the disease, syphilis typically enters its latent phase, during which no clinical symptoms are present.

Late syphilis, in its latent late form, may persist asymptomatically for many years. Rarely, secondary symptoms may reoccur during this stage [4,5,6]. Late syphilis manifests clinically through organ-specific damage, depending on the systems involved. There are three main forms of late syphilis, which are as follows: gummatous syphilis, neurosyphilis, and cardiovascular syphilis. In the authors’ experience, it is not uncommon for patients to be tested for syphilis only after the appearance of neurological, psychiatric, or ophthalmological symptoms, which eventually lead to the correct diagnosis. The symptoms of late syphilis are often nonspecific and may be inconsistent with laboratory findings, making its diagnosis particularly challenging.

The ideal clinical case involves the patient going to the clinician after a single risky occurrence of sexual behaviour with a particular date, after some symptoms of early syphilis occur. The diagnosis is easy, and the treatment may be introduced early and according to the stage of the disease.

The problem with patients with syphilis is that some of them do not know when the disease started due to their engagements in risky sexual behaviours that date years back. Moreover, during the period of late syphilis, such patients may be reinfected with syphilis, potentially developing the symptoms of the early stages of disease. Every person that is sexually active should be regularly screened for at least the following: syphilis, HIV, and HBV with HCV [1,2,3].

Treponemal and non-treponemal tests are used in the diagnosis of syphilis. Screening diagnostics can be performed using treponemal tests (TPHA or EIA/ELISA/CLIA), which identify both currently infected patients and those who have successfully completed treatment, but they are more sensitive in detecting early syphilis. Non-treponemal tests (RPR/VDRL) are still widely used in many countries and detect only active forms of the disease, with slightly lower sensitivity than treponemal tests. To confirm the diagnosis, it is necessary to obtain two positive test results—preferably one non-treponemal and one treponemal. For further management, determining the RPR/VDRL titer is important, as it allows for the monitoring the effectiveness of treatment [1,2,3].

Treatment of syphilis depends on the stage of the disease. In general, regimens based on long-acting benzathine penicillin G are recommended whenever possible. However, alternative treatments may be used in cases of allergy, unavailability of penicillin, or when the patient refuses parenteral administration.

After administering the syphilis treatment appropriate to the stage of the disease and its symptoms, it is very important to monitor the effectiveness of the therapy. This involves assessing the RPR or VDRL titer at 1, 3, and 6 months after treatment, and then every 6 months thereafter. After the treatment of early syphilis, the titre of RPR/VDRL taken on day 0 should decline by ≥2 dilution steps (fourfold decrease in titre of antibodies) within 6 months. Then, patients should be monitored until the satisfactory low titers are reported.

Effective treatment is indicated by either a negative test result or a sustained low titer plateau (1:1–1:4). The monitoring of low titers can be stopped and the patient can be considered cured after at least one to two years of observation. However, some patients do not achieve a satisfactory decrease in titer. This may be due to as yet unidentified reasons, where an effectively treated patient fails to serorevert and is considered serofast. However, asymptomatic neurosyphilis must first be ruled out.

Patients undergoing treatment are recommended to inform their sexual partners about the need for diagnostic testing and, if necessary, treatment, as well as the importance of using effective barrier methods to prevent the transmission of the disease until a cure is confirmed.

Monitoring serological titers is crucial for identifying possible treatment failure or reinfection. To control the spread of the disease, testing should not be limited to individuals already affected. Syphilis is a disease that can affect any sexually active person. Accessible screening and professional consultation in a comfortable setting are essential for effective disease control. Each case requires individual assessment based on laboratory findings and a thorough patient history.

We present the case of a patient who underwent syphilis testing due to a history of regular high-risk sexual behaviour over the past several years. He sought screening for sexually transmitted infections after noticing new skin lesions. The appearance of the lesions was not typical for syphilis, and it was only the patient’s history of high-risk behaviours that prompted further investigation, ultimately leading to the correct diagnosis. It was not possible to determine with certainty which stage of the disease should be diagnosed. Although recurrent skin lesions were suggestive of early latent syphilis, the exact timing of infection could not be determined. As it was presumed to have occurred more than one year prior to presentation, the patient was managed as a case of late latent syphilis.

## 2. Case Report

**Figure 1:**
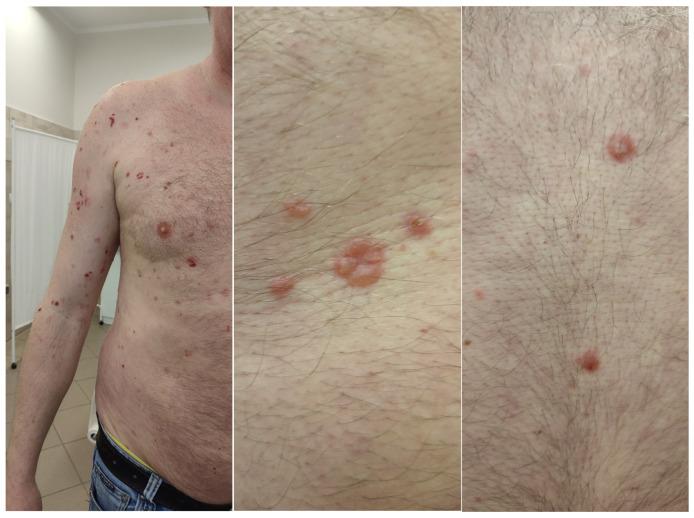
Polymorphic, vesiculobullous lesions.

A 46-year-old man presented to a venerology clinic with a polymorphic skin rash—comprising erythematous macules, vesicles, and bullae—that had persisted for several weeks (Figure 1). He did not notice any lesions on the genital area or mucose membranes, and none were found during the physical examination. His medical history included arterial hypertension, alcohol and sex addiction, and sexual behaviour with multiple partners of both sexes annually. He had previously been treated empirically for urethral discharge without medical documentation.

Serological tests confirmed Treponema pallidum infection. TPHA and WR (Wassermann test) were positive, with a VDRL titer of 1:128. HIV, anti-HCV, and HBsAg were negative. He was diagnosed with late syphilis because of the unknown duration of the disease. Intramuscular benzathine penicillin was advised as first-line therapy; however, the patient refused injections. Oral doxycycline (100 mg BID for 28 days) was initiated but delayed by 12 days due to difficulty with alcohol cessation.

**Figure 2:**
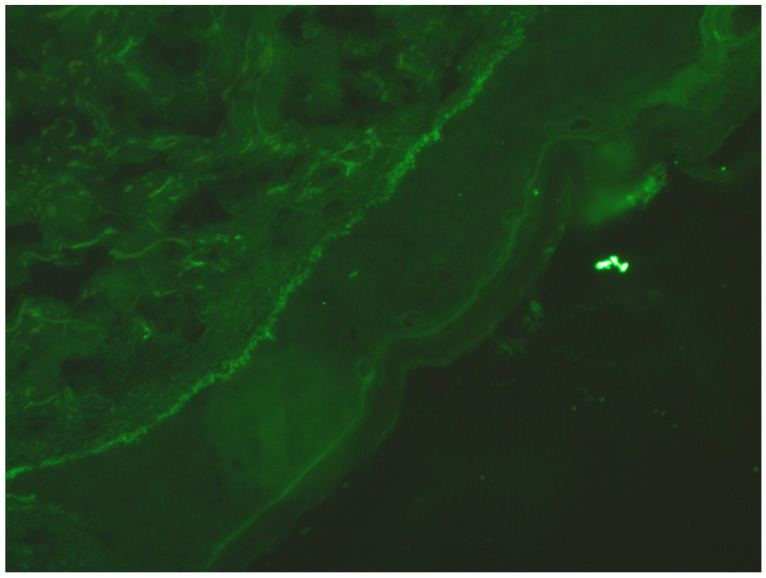
Direct immunofluorescence FIG- C1q deposits in dermoepidermal junction.

Twenty days into treatment, he returned with worsening erosive and vesiculobullous lesions and was hospitalised. Laboratory findings revealed elevated CRP (42.3 mg/L), a low neutrophil count (33.1%), and a high lymphocyte count (53.3%). A rheumatologic consultation suggested possible reactive arthritis due to left knee pain, but this was excluded at the end. A skin biopsy for direct immunofluorescence (DIF) was negative for bullous dermatoses, showing only nonspecific C1q deposition (Figure 2). The histopathological report from the biopsy described subcorneal and subepidermal blisters with inflammatory infiltration consisting of lymphocytes, eosinophils, and neutrophils, localised in and under the epidermis and around skin appendages. No plasmacytes were observed. Serologic tests for pemphigus/pemphigoid antibodies, Chlamydia IgM/IgG, Yersinia IgM/IgG and urine Chlamydia PCR were all negative. No neurological or ophthalmological symptoms were noted.

As the previously settled diagnosis was not changed due to negative tests for bullous autoimmunologic diseases, during hospitalisation, intramuscular benzathine penicillin (2.4 million IU weekly × 1-8-15) replaced doxycycline, resulting in skin improvement.

Eight weeks post-treatment, the patient reappeared at the outpatient clinic with new, transient vesicular lesions and a persistent VDRL titer of 1:128. The next VDRL testing was planned after four weeks, but the patient did not appear.

**Figure 3:**
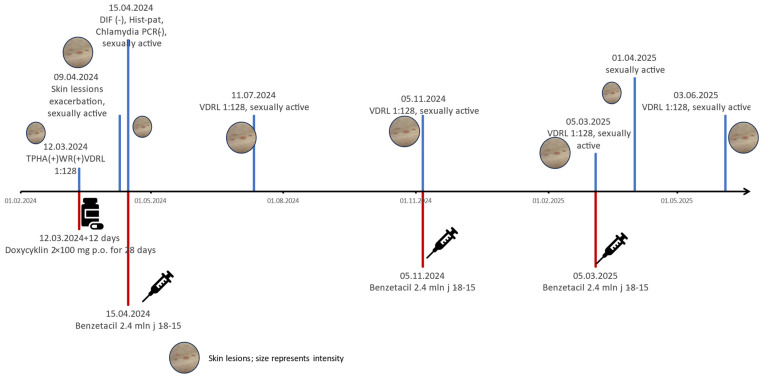
Patient’s temporal axis of disease. Color blue and red are supposed to differentiate the treatment form lab test.

Four months later, he returned with similar bullous and vesicular lesions, preceded by symptoms of upper respiratory tract infection. He admitted to engaging in unprotected sex with multiple partners after the prior treatment, suggesting reinfection. New serologic and immunologic tests were performed, including desmoglein antibodies, tissue transglutaminase (IgA/IgG), second DIF, second ANA/HEp-2, and HIV, which were negative. VDRL remained at 1:128. A second course of benzathine penicillin (3 × 2.4 million IU) was administered as an outpatient after the patient declined hospitalisation. After the first dose of the benzathine penicillin was given, the patient did not come for the second injection on time. He came a few weeks later, and then the entire cycle of 1-8-15 2.4 mL of benzathine penicillin was completed. He later missed the first serological test and returned a few months later with a titer of VDRL 1:128. He admitted engaging in risky sexual behaviour and agreed to the thesis of next reinfection.He noticed improvement in the skin lesions after treatment but observed new lesions after potentially risky behaviours. However, the ineffectiveness of the therapy must also be considered. He was scheduled for hospitalisation to exclude central nervous syphilis and complete the treatment with crystalline penicillin but he did not agree. The patient was reported to the institution responsible for control and enforcement of the treatment of sexually transmitted diseases in order to ensure appropriate epidemiological supervision, implement mandatory treatment, and reduce risk of further transmission of the infections. He was informed that only the discontinuation of risky sexual behaviour, thereby eliminating the risk of reinfection, would allow for the further monitoring and effective management of the disease (Figure 3).

## 3. Discussion

Syphilis is a disease of protean manifestations. The screening test may be performed because of typical symptoms and a history of risky sexual behaviour in the patient, with or without symptoms, but may also be performed in patients with symptoms that do not fit any other disease.

Vesicles and bullas that last for weeks and are disseminated are usually a manifestation of autoimmunological blistering diseases. Patients may present lesions on the skin and mucose membranes. Diagnosis of such diseases is based on the direct immunofluorescence test (DIF) from a biopsy taken from healthy skin next to the lesion and/or serum anty-pemfigus/pemfigoid antibodies or the ELISA test. The treatment consists of immunosuppressive drugs, depending on the exact diagnosis.

In this case, the unusual vesiculobullous rash prompted extensive differential diagnostics. Even though the patient came to the outpatient clinic with a positive screening test, the skin lesions were not typical for syphilis and the coexistence of other disease had to be excluded.

Our patient had negative DIF and serology for autoantibodies, which helped exclude autoimmune blistering diseases, such as pemphigus vulgaris, bullous pemphigoid, or dermatitis herpetiformis. Therefore, the lesions were considered an atypical manifestation of syphilis.

Although vesicles and bullas are typical in congenital syphilis [13], they are rarely seen in syphilis in adults.

There are few reports of syphilis presenting with bullous and/or vesicular lesions. The resemblance of skin lesions to bullous diseases made all the authors first look for autoimmunologic blistering disorders. Some even started the treatment with immunosuppressants. However, the immunological testing (DIF and serum desmoglein antibodies) were inconsistent in different patients. There is no common pattern of immunological phenomena in the skin of affected patients.

Stone et al. [14] and Mignogna et al. [15] presented case reports of patients with bullous lesions and positive DIF, characteristic of pemphigus. Lawrence [16] presented a case report of a patient with positive DIF, similar to pemphigoid. Kopelman at al. [17] presented a case of a patient with negative both DIF and antibodies levels, results similar to those described in this paper’s patient. Pacheco [18] described similar bullous eruptions with negative DIF but positive IgG desmoglein-1 and borderline/indeterminate desmoglein-3 antibody levels. Other cases were reported by Arora et al. [19] and Lourari et al. [20], where patients were diagnosed with syphilis and no blistering diseases laboratory tests were performed, but in those patients HIV infection was confirmed. Shnirring-Judge et al. reported a case report of syphilis manifesting with large bullous lesion on the feet, and no histopathology nor immunohistology was performed in this case. The lesions in all reported patients improved after syphilis treatment. In our patient, it is currently not possible to assess the effectiveness of treatment due to ongoing risky behaviours that may lead to reinfection, with no reduction in VDRL titer observed so far (Table 1).

The role of immunological findings in the context of *Treponema pallidum* infection accompanied by bullous lesions remains unclear. Although only a small number of such cases have been reported, older texts in venerology do describe these unusual presentations. The reason why some patients develop bullous lesions is still not well understood.

However, reported cases indicate that blisters may form in different layers of the skin—ranging from the epidermis to the dermoepidermal junction—implying that these lesions may not be due to individual predisposition alone. An alternative explanation for this rare manifestation could lie in specific characteristics of the *Treponema pallidum* strain itself. In this regard, the genetic mapping of *T. pallidum* directly from tissue samples could offer valuable insights into the pathogenesis of these unusual cutaneous features.

## 4. Conclusions

We present a rare case of recurrent vesiculobullous syphilis mimicking autoimmune dermatoses, discussed in the context of other similarly reported cases. Atypical skin manifestations—particularly when accompanied by inconsistent serological results—should prompt clinicians to consider syphilis in the differential diagnosis. After all, syphilis is aptly known as “the great imitator”.

This case highlights the importance of accessible syphilis screening, which is essential for identifying non-classical or atypical presentations. Additionally, the report underscores diagnostic challenges, treatment barriers, and the broader public health implications of managing high-risk individuals with poor treatment adherence.

## Figures and Tables

**Table 1:** Reported patients with secondary syphilis and vesicles/bullous lesions.

	Age	Sex	Direct Immunofluorescence	Histopathology	Antibodies Anti-Desmoglein 1 or 3	Treatment
Stone et al. [14]	60	male	granular IgG on the surface of epithelial cell continuous in multiple areas but seldom covered the entire epithelial cell surface	parakeratosis with inflammatory cell debris overlying an epidermis manifesting focal intraepithelial acantholysis involving follicle, superficial and deep perivascular and interstitial nodular infiltrate composed of lymphocytes with large numbers of plasma cells and a few scattered eosinophils, histiocytes and neutrophils	not done	penicillin G 24 mln U/day iv 14 days—complete resolution of skin lesions
Mignogna et al. [15]	47	female	positive for intercellular cement substance (ICS), with its classic ‘net-like’ aspect	suprabasal epithelial detachment with a prominent eosino-neutrophilic infiltrate, suggesting a possible PV	negative	benzathine penicillin G twice a week 1.2 mln U im with skin lesions resolution in 4 weeks
Lawrence et al. [16]	40	male	linear IgG and C3 at the basement membrane	blister with uni-locular subepidermal vesicle with perivascular lymphocytic infiltrate and occasional eosinophils and plasma cells in upper-dermis	negative	procaine penicillin for 10 days with skin lesions resolution
Kopelman et al. [17]	18	male	negative	subcorneal acantholytic vesicle containing neutrophils. The underlying superficial dermis contains a perivascular infiltrate of lymphocytes, neutrophils, and occasional eosinophils	negative	benzathine penicillin G of one dose with skin lesions resolution
Pacheco [18]	52	female	no data	lichenoid and perivascular dermatitis with numerous plasma cells, negative immunostaining for spirochetes	positive IgG desmoglein-1 and borderline/indeterminate desmoglein-3 antibody levels	benzathine penicillin G im 1-8-15 with skin lesion resolution with hyperpigmentation
Arora et al. [19]	45	male	not done	lymphohistiocytic infiltrate and plasma cells	Not done	benzathine penicillin G 2.4 mln im 1-8-15 with skin lesions resolution
Lourari et al. [20]	45	male	not done	focally ulcerated epidermis, beneath which developed an inflammatory lesion rich in mature plasma cells, extending through the entire thickness of the dermis down to the hypodermis	Not done	benzathine penicillin 2.4 mln U im 1-8-15 with skin lesions resolution
Schnirring-Judge et al. [21]	41	male	not done	not done	Not done	benzathine penicillin G 2.4 mln U im once with skin lesions resolution
Described patient	46	male	negative	subcorneal and subepidermal blisters with inflammatory infiltration consisting of lymphocytes, eosinophils, and neutrophils, localised in and under the epidermis and around skin appendages. No plasmocytes we observed.	negative	benzathine penicillin G 2.4 mln U im 1-8-15 but because of no decrease in VDRL titers (reinfection?) the cycle was repeated 3 times

## Data Availability

The data presented in this study are available on request from the corresponding author due to general data protection regulation.

## References

[1] Janier M., Unemo M., Dupin N., Tiplica G.S., Potočnik M., Patel R. (2021). 2020 European guideline on the management of syphilis. J. Eur. Acad. Dermatol. Venereol..

[2] European Centre for Disease Prevention and Control http://www.ecdc.europa.eu.

[3] World Health Organization (2016). WHO Guidelines for the Treatment of *Treponema pallidum* (*Syphilis*). https://www.who.int/publications/i/item/who-guidelines-for-the-treatment-of-treponema-pallidum-(syphilis).

[4] Miedziński F. (1985). Choroby Skóry i Weneryczne.

[5] Jabłońska S. (1967). Choroby Weneryczne.

[6] Walter F. (1950). Choroby Weneryczne.

[7] Hira S.K., Patel J.S., Bhat S.G., Chilikima K., Mooney N. (1987). Clinical manifestations of secondary syphilis. Int. J. Dermatol..

[8] Balagula Y., Mattei P.L., Wisco O.J., Erdag G., Chien A.L. (2014). The great imitator revisited: The spectrum of atypical cutaneous manifestations of secondary syphilis. Int. J. Dermatol..

[9] Noppakun N., Dinehart S.M., Solomon A.R. (1987). Pustular secondary syphilis. Int. J. Dermatol..

[10] Sun Y., Zhao W., Li F., Chen S., Tian H. (2025). Isolated initial clavus-like rash: A rare presentation of secondary syphilis. Int. J. STD AIDS.

[11] Dourmishev L.A., Dourmishev A.L. (2005). Syphilis: Uncommon presentations in adults. Clin. Dermatol..

[12] Stone C.E., Onyekaba N.A., Lucas M., Jukic D. (2020). Cutaneous Secondary Syphilis Resembling Non-Melanoma Skin Cancer. Cureus.

[13] Kim J.K., Choi S.R., Lee H.J., Kim D.H., Yoon M.S., Jo H.S. (2011). Congenital syphilis presenting with a generalized bullous and pustular eruption in a premature newborn. Ann. Dermatol..

[14] Stone C.J., Nicholson L., Florell S.R., Khalighi M.A., Lewis B.K.H. (2023). A case of secondary syphilis presenting like pemphigus with positive direct immunofluorescence. JAAD Case Rep..

[15] Mignogna M.D., Fortuna G., Leuci S., Mignogna C., Delfino M. (2009). Secondary syphilis mimicking pemphigus vulgaris. J. Eur. Acad. Dermatol. Venereol..

[16] Lawrence P., Saxe N. (1992). Bullous secondary syphilis. Clin. Exp. Dermatol..

[17] Kopelman H., Lin A., Jorizzo J.L. (2019). A pemphigus-like presentation of secondary syphilis. JAAD Case Rep..

[18] Pacheco C.C., Sinha S., Sloan B., Whitaker-Worth D., Murphy M.J. (2024). Comments on Stone et al, “A case of secondary syphilis presenting like pemphigus with positive direct immunofluorescence”. JAAD Case Rep..

[19] Arora S., Dhali T.K., Haroon M.A. (2013). Vesicular syphilid in a seropositive patient. Int. J. STD AIDS.

[20] Lourari S., Bulai-Livideanu C., Giordano-Labadie F., Lamant L., Launay F., Viraben R., Paul C. (2011). Syphilis secondaire bulleuse [Bullous secondary syphilis]. Presse Med..

[21] Schnirring-Judge Molly A., Gustaferro C., Terol C. (2011). Vesicobullous syphilis: A case involving an unusual cutaneous manifestation of secondary syphilis. J. Foot Ankle Surg..

